# Clinical utility of a novel test for assessing cardiovascular disease risk in type 2 diabetes: a randomized controlled trial

**DOI:** 10.1186/s13098-023-01122-w

**Published:** 2023-07-13

**Authors:** John W. Peabody, David Paculdo, Enrico de Belen, Divya Ganesan, Isabella Cooney, Nelson Trujillo

**Affiliations:** 1 QURE Healthcare, 450 Pacific Ave., Suite 200, San Francisco, CA 94133 USA; 2 SomaLogic Operating Co., Inc., 2945 Wilderness Pl., Boulder, CO USA; 3 University of California, San Francisco, 550 16th Street, Third Floor, San Francisco, CA USA; 4 University of California, Los Angeles, 650 Charles E. Young Dr S, Los Angeles, CA USA; 5 Boulder Community Health, 4747 Arapahoe Ave., Boulder, CO USA

**Keywords:** Primary care, Cardiometabolic disease, Polypharmacy, Drug-drug interactions, Medication nonadherence, Disease progression, Clinical utility

## Abstract

**Background:**

The risk for and treatment of cardiovascular disease (CVD) in type 2 diabetes (T2DM) is often incorrect and delayed. We wished to determine if a novel test improved physicians’ ability to risk stratify, diagnose, and treat patients with T2DM.

**Methods:**

In a 2-phase randomized controlled trial comparing the clinical workup, diagnosis, and management of online, simulated patients with T2DM in a nationwide sample of cardiologists and primary care physicians, participants were randomly assigned to control or one of two intervention groups. Intervention participants had access to standard of care diagnostic tools plus a novel diagnostic CVD risk stratification test.

**Results:**

In control, there was no change in CV risk stratification of simulated patients between baseline and round 2 (37.1 to 38.3%, p = 0.778). Pre-post analysis showed significant improvements in risk stratification in both Intervention 1 (38.7 to 65.3%) and Intervention 2 (41.9 to 65.8%) (p < 0.01) compared to controls. Both intervention groups significantly increased prescribing SGLT2 inhibitors/GLP1 receptor agonists versus control, + 18.9% for Intervention 1 (p = 0.020) and 1 + 9.4% for Intervention 2 (p = 0.014). Non-pharmacologic treatment improved significantly compared to control (+ 30.0% in Intervention 1 (p < 0.001) and + 22.8% in Intervention 2 (p = 0.001). Finally, monitoring HgbA1C, blood pressure, and follow-up visit frequency improved by + 20.3% (p = 0.004) in Intervention 1 and + 29.8% (p < 0.001) in Intervention 2 compared with control.

**Conclusion:**

Use of the novel test significantly improved CV risk stratification among T2DM patients. Statistically significant increases treatments were demonstrated, specifically SGLT2 inhibitors and GLP1 receptor antagonists and recommendations of evidence-based non-pharmacologic treatments.

*Trial registration* ClinicalTrials.gov, NCT05237271

**Supplementary Information:**

The online version contains supplementary material available at 10.1186/s13098-023-01122-w.

## Background

Cardiovascular disease (CVD) is the most prevalent cause of death in both type 1 and type 2 diabetes mellitus (DM) [1–3]. Compared to patients without DM, the relative risk for CVD morbidity and mortality in adult diabetics ranges from 1 to 3 in men and from 2 to 5 in women [4]. Increasingly understanding of the traditional CVD risk factors of hyperglycemia, obesity, smoking, hypertension, and dyslipidemia are mediated by increased oxidative stress, hypercoagulability, and endothelial dysfunction, which lead to the development of CVD [2]. Indeed, obesity itself is associated with low-grade systemic inflammation and predicts the development of type 2 diabetes [5]. Multiple biomarkers are associated with CVD and type 2 diabetes, such as angiopoietin 2 and matrix metalloproteinase 12, to name a few [6, 7].

Several studies, including the Steno-2 trial suggest control of multiple cardiovascular (CV) risk factors in patients with type 2 diabetes mellitus (T2DM) can decrease the risk of CV events and mortality by half [8]. Other studies show optimal control of these risk factors is not easily achieved, with only about 20–25% of T2DM patients at the goal for blood pressure, glycated hemoglobin, and low-density-lipoprotein cholesterol targets [9, 10]. Long standing reports document the importance of providers advising their patients to diet, exercise and quit smoking [11–13]. More recently investigators have found use of novel glycemia-lowering therapies with cardioprotective features remains profoundly low (< 10% of eligible patients) despite proven efficacy, professional society guideline endorsement, and regulatory labels for CV benefit [14].

Due to the complex interplay between T2DM and CVD and the potential impact of counselling and pharmacologic therapy by clinicians, it is essential that each DM patient’s risk be characterized. Among primary care physicians (PCPs) and cardiologists, however, there is widespread variation in using stratification tools and accuracy of CVD risk calculation in patients with T2DM. In our study study, in a group of 241 clinicians, 18.3% of cases were not risk stratified and of the remainder, 42.8% of cases had the CV risk incorrectly stratified [15]. A better diagnostic tool, more focused on the individual’s aggregate CVD risks might help guide physicians in streamlining evidence-based interventions to achieve target goals.

SomaLogic Operating Co., Inc developed a pioneering technology, the SomaScan® Platform, that simultaneously measures 7000 proteins with high sensitivity and specificity in one serum sample. Using over 60,000 samples, artificial intelligence, and machine learning powered bioinformatics algorithms, the company created the CardioDM Test, [also known as the Cardiovascular Disease in Type 2 Diabetes (CVD-T2D) Test], which produces an individual’s risk score for developing cardiovascular events such as heart attacks, strokes, hospitalization for heart failure, or cardiovascular death within 4 years [16]. The CVD-T2D test has the potential to effectively risk-stratify patients, determine disease prognosis, and improve patient outcomes.

The primary objective of this study was to determine if the CVD-T2D Test leads to an improvement in the ability to risk stratify, diagnose, and treat patients with T2DM.

## Methods

### Ethics

The study was conducted in accordance with ethical standards, approved by Advarra Institutional Review Board, Columbia, MD, and registered on clinicaltrials.gov (NCT05237271). Informed consent was obtained from all participants through an online voluntary consent process. All data were kept confidential.

### Study description and design

The QURE CVD Evaluation of Risk in Diabetes Mellitus (QuiCER DM) Study was conducted between March and June 2022 using Clinical Performance and Value (CPV®) simulated patient cases, comparing the clinical workup, diagnosis, and management of patients with T2DM. Participants were randomly assigned to control, intervention with only the CVD-T2D Test, or a second intervention which used both the CVD-T2D Test and a Metabolic Factors Panel. We used a pre post two-round design and each participant cared for six simulated CPVs, three in each round.

### Clinical performance and value (CPV®) vignettes

The CPV randomized controlled trial is a proven methodology widely used to rapidly measure physician care decisions [17]. CPVs are a uniquely validated and scalable tool that standardizes practice measurement by having all providers care for the same (virtual) patients [18]. With all providers caring for the same patients, the CPVs generate unbiased data that yields powerful insights into clinical decision-making and how these decisions change with the introduction of a new product or solution [19]. CPVs are a peer-reviewed and validated measure of clinical practice [20–24] and use open-ended questions simulating typical patient encounters, with questions divided into five domains of care: (1) history taking, (2) physical examination, (3) diagnostic work-up, (4) making a diagnosis with CV risk stratification, and (5) management plan and monitoring.

Between 53 and 61 evidence-based criteria were evaluated for each CPV based on best practice and current guidelines, such as the Standards of Medical Care in Diabetes by the American Diabetes Association (ADA). Two trained expert physicians working independently scored the responses of the participants using these pre-determined criteria. In case of disagreement, a third physician would serve as an adjudicator for the final score. A quality-of-care score (ranging from 0 to 100%) was generated in each clinical domain of care. Higher scores indicated greater adherence to the evidence base in clinical care provided.

### SomaSignal™ test

The CVD-T2D test utilizes a single blood sample and has been validated in a broad population of diabetic patients including patients with multiple comorbidities, and across and range of ethnicities and socioeconomic groups, to provide an individualized patient-specific risk score [16]. The test estimates the absolute risk of having a cardiovascular event (myocardial infarction, stroke, heart failure-related hospitalization, all-cause death) within 4 years for patients over 40 years of age, with type 2 diabetes, with or without cardiovascular disease (CVD) or chronic kidney disease. The range of scores is from 0 to 100%. A score between 50 and 100% is high risk, 25 to 49% medium high risk, 7.5 to 24% is medium low risk, and less than 7.5% is low risk. The accompanying Metabolic Panel, measured from the same blood sample, measures the likelihood of liver fat and impaired glucose tolerance, estimated glomerular filtration rate (eGFR), alcohol impact, peak exercise capacity, resting energy rate, body fat percentage, lean body mass, and visceral fat.

### Physician recruitment

From a nationally representative roster of 12,500 physicians, PCPs and cardiologists were sequentially invited to participate in the study. Physicians were eligible if they met the following criteria: (1) board-certified in family medicine, internal medicine, or cardiology for at least 2 years, (2) averaged at least 20 h per week of clinical and patient care duties over the last 6 months, (3) routinely evaluated patients with T2DM in their practice, (4) naïve to the CVD-T2D test, (5) practiced in the United States, (6) English-speaking, (7) had internet access, and (8) voluntarily gave informed consent to participate. After screening for eligibility, we originally enrolled 161 PCPs and 80 cardiologists achieving a 2:1 ratio. Among this group, eight PCPs and five cardiologists did not complete their participation and were excluded from the study. The final provider roster was then randomized into one control group and two intervention groups: Intervention 1 and Intervention 2.

### Intervention

**Fig. 1:**
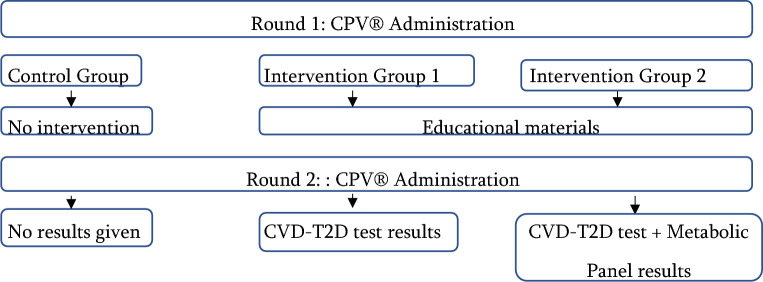
Study design

All providers were naïve to the CVD-T2D test and the Metabolic Panel at baseline. Two to three weeks after baseline CPV data collection, Intervention 1 physicians received educational materials on the CVD-T2D test and Intervention 2 physicians received educational materials on the CVD-T2D test and the Metabolic Panel (see Additional files 2 and 3 for greater details on these tests). The educational material consisted of a slide deck and fact sheet on the CVD-T2D test ± Metabolic Panel. Intervention participants could not complete the study without first reviewing these materials. All providers were then asked to complete three new CPVs (with similar characteristics as those in the first round) approximately 2 weeks after the intervention groups reviewed their education materials. In this second round of data collection, Intervention 1 participants were provided the CVD-T2D test result, while Intervention 2 participants were given the CVD-T2D test results together with the Metabolic Panel results. Participants in the control arm had access to standard of care diagnostic tools, but not the CVD-T2D test or the Metabolic Panel results (Fig. 1).

### Specific CPV patient cases

We developed three types of patients to determine the potential impact of the CVD-T2D test without and with the Metabolic Panel compared to the controls: (1) patients with T2DM who had 3 or more CV risk factors, (2) patients with T2DM with 2 CV risk factors, and (3) patients with T2DM and CKD. For each of the three case types, three case variants were created to investigate the clinical utility of the novel risk stratification tool: Case variant A involved patients with high clinical CV risk and a high-risk CVD-T2D test result. Case variant B involved patients with intermediate clinical CV risk and a high-risk CVD-T2D test result. Case variant C involved patients with high clinical CV risk and a low-risk CVD-T2D test results. These cases are summarized in Additional file 1: Table S1.

### Data collection tools

Our study used two sources of data: (1) a physician survey to obtain demographic background information for each participant and (2) the scored CPV vignettes. The participant survey included questions on age, gender, specialty, practice type, location and setting of practice, and payer mix. Physicians were also asked if they used a CV risk stratification tool in their practice.

### Study outcomes and statistical analysis

The primary outcome was whether use of the CVD-T2D test ± Metabolic Panel, demonstrated greater clinical utility and improved patient care. After controlling for provider and clinical practice characteristics, we wanted to determine if there were improvements in (1) correct CV risk stratification rates after the providers received the CVD-T2D test results and (2) the rates of interventions provided to target modifiable CV risk factors to attain appropriate evidence-based goals, including pharmacologic, non-pharmacologic (referrals, counseling), and monitoring/follow-up.

Summary statistics were determined for all variables. Numerical variables were summarized through mean and standard deviation or median and interquartile range. We used the Chi-squared test and logistic regression to analyze binary outcomes. Our study was sufficiently powered to detect a 5% difference in clinical variables with an alpha of 0.05 and power of 80%. All analyses were performed using Stata 17.0 (StataCorp LLC, College Station, TX).

## Results

### Physician characteristics

228 physicians participated in the study, with no significant differences between the three study arms (Table 1). Intervention 1 had fewer cardiologists (24.3%) compared to the other arms (38.8% and 36.5% for control and intervention 2, respectively); control were slightly older than either intervention arm (58.4 years for control versus 55.1 years for intervention 1 and 56.9 years for intervention 2); but these did not achieve significance (p > 0.05 for both). Nearly 80% of participants were male; over half of the physicians practiced in a suburban setting, and over 80% were employed by their practice. Most physicians also claimed to use a cardiovascular risk calculator in their practice.

**Table 1:** Physician characteristics

	Control	Intervention 1	Intervention 2	p-value
N	80	74	74	–
Male	77.5%	82.4%	74.3%	0.486
Cardiology	38.8%	24.3%	36.5%	0.129
Age (years)	58.4 ± 8.7	55.1 ± 8.8	56.9 ± 9.9	0.079
Practice type				
Academic	2.5%	1.4%	4.1%	0.984
Hospital-based	13.8%	10.8%	13.5%	
Private, multi-specialty	26.3%	29.7%	28.4%	
Private, single specialty	37.5%	35.1%	39.2%	
Private, solo	15.0%	18.9%	12.2%	
FQHC	3.8%	2.7%	2.7%	
Other	1.3%	1.4%	0.0%	
Region				
Northeast	22.5%	35.1%	40.5%	0.118
South	23.8%	24.3%	24.3%	
Midwest	30.0%	20.3%	12.2%	
West	23.8%	20.3%	23.0%	
Setting				
Urban	30.0%	36.5%	32.4%	0.803
Suburban	56.3%	55.4%	55.4%	
Rural	13.8%	8.1%	12.2%	
Employed by practice	86.3%	87.8%	83.8%	0.774
Payer, %				
Medicare	38.6%	33.9%	38.8%	0.120
Medicaid	11.3%	10.1%	12.7%	0.496
Commercial	45.6%	50.5%	43.5%	0.068
Self	3.7%	4.1%	4.0%	0.835
Other	0.8%	1.3%	1.0%	0.599
Use CV risk calculator	80.0%	83.8%	81.1%	0.825

### Changes in diagnostic risk stratification

In control, we found no significant change in cardiovascular risk stratification from baseline round 1 to round 2 (37.1 to 38.3%, p = 0.778). By contrast, there were significant improvements in risk stratification in both Intervention 1 (38.7 to 65.3%) and Intervention 2 (41.9 to 65.8%) across all cases. The formal difference-in-difference estimation model showed improvements of + 25.4% and + 22.7% for intervention 1 and intervention 2 compared to control (p < 0.01 for both). By case type, the most significant improvement was in patients with 3+ CV risk factors (Table 2), where Intervention 1 had a difference-in-difference improvement of + 26.3% (p = 0.020) and Intervention 2 had a difference-in-difference improvement of + 27.7% (p = 0.015), both compared to Control.

**Table 2:** Risk stratification

	Round 1	Round 2	p-value
All cases			
Control	37.1%	38.3%	0.778
Int 1	38.7%	65.3%	< 0.001
Int 2	41.9%	65.8%	< 0.001
p-value	0.564	< 0.001	
3+ CV risk factors			
Control	37.5%	28.8%	0.240
Int 1	43.2%	60.8%	0.032
Int 2	40.5%	59.5%	0.021
p-value	0.768	< 0.001	
2 CV risk factors			
Control	28.8%	46.3%	0.022
Int 1	29.7%	70.3%	< 0.001
Int 2	31.1%	71.6%	< 0.001
p-value	0.951	0.001	
Diabetic kidney disease			
Control	45.0%	40.0%	0.522
Int 1	43.2%	64.9%	0.008
Int 2	54.1%	66.2%	0.131
p-value	0.366	0.001	

*CV* cardiovascular

### Changes in treatment

Next, we looked at how frequently physicians provided treatment and follow-up for their patients. We divided treatment into pharmacologic, non-pharmacologic, and follow-up care to see if test use increased physician engagement in discussing these issues with the patient or prescribing necessary drug treatments. For non-pharmacologic care, we measured whether the participants advised their patients on at least half of the following care elements: advising on nutrition, weight, exercise, smoking, drinking, etc. or referrals to specialists, anywhere from five to seven items depending on patient. Similarly, for pharmacologic treatment, we measured five to eight items: whether providers added, continued, switched, or stopped diabetes- (metformin, insulin, sulfonylurea, SGLT2i, GLP1 RA) and CV- (statin, antihypertensive) related drugs at least half the time.

For pharmacologic care, we found no significant differences between the Control and Intervention groups between rounds 1 and 2. Overall, participants at baseline performed more than half of the pharmacologic treatment items in only 7.8% of cases. A full regression model on the baseline data, which accounted for physician demographics and practice, showed no significant difference between either Intervention group or Control (p > 0.05 for both). We found no differences in either intervention arm compared to control in the pre-post analysis (Intervention 1: OR 0.8, 95% CI 0.3–2.5; Intervention 2: OR 0.8, 95% CI 0.4–2.0), when we repeated the full logistic analysis (Table 3a). When we looked, however, at prescribing SGLT2 inhibitors and GLP1 receptor agonists, the intervention arms improved significantly between rounds 1 and 2, with difference-in-difference improvements of + 18.9% for Intervention 1 (p = 0.020) and + 19.4% for Intervention 2 (p = 0.014), both compared against Control. (Table 4). Intervention physicians were also more likely to order these medications for their T2DM patients if they had kidney disease and 2 CV risk factors than those with 3+ CV risk factors (p < 0.001). Importantly, SomaSignal scores of > 50% were associated with significant increases in utilizing SGLT2 inhibitors and GLP1 receptor agonists. In simulated patients with very high scores, i.e., > 90%, prescribing improved from 41 to 68% in Intervention 1 (p = 0.001) and from 49 to 72% in Intervention 2 (p = 0.004). Similarly, in the lower end of the high-risk scores, i.e., 55 to 80%, prescribing evidence-based therapies increased from 38 to 68% and 39 to 73% in Interventions 1 and 2, respectively (p < 0.001 for both). Low SomaSignal scores (< 20%) did not change prescribing habits. Surprisingly, correct use of moderate or high intensity statins did not increase with correct CV risk stratification. However, statin ordering overall was significantly higher for both intervention groups with Intervention 1 2.0x (95% CI 1.1–3.4) and Intervention 2 2.1x (95% CI 1.2–3.6) after introduction of the test compared to Control. Antihypertensive ordering did not improve for intervention, either individually or combined. When we conditioned pharmacologic treatment on correct CV risk assessment, we found no significant improvement, with both Intervention 1 and Intervention 2 doing nonsignificantly worse round-to-round compared to control, − 3.7% and − 4.3%, respectively (p > 0.05 for both).

**Table 3:** Logistic regression of participants ordering a majority of pharmacologic treatment, non-pharmacologic treatment, and monitoring/follow-up

(a) Pharmacologic treatment				
	OR	95% CI		P > z
		Lower	Upper	
Male	0.56	0.36	0.89	0.014
Age < 45	0.53	0.27	1.06	0.074
Cardiologist	1.00	0.63	1.59	0.999
South region	0.73	0.45	1.19	0.211
Rural	2.18	1.32	3.62	0.003
Academic practice	2.04	0.73	5.72	0.174
Study arm				
Intervention 1	0.47	0.21	1.06	0.070
Intervention 2	1.14	0.61	2.14	0.680
Round	1.32	0.73	2.38	0.367
Study arm * round				
Intervention 1 * round 2	0.85	0.28	2.55	0.771
Intervention 2 * round 2	0.84	0.36	1.98	0.689
Constant	0.15	0.08	0.25	0.000

(b) Non-pharmacologic treatment				
Nonpharmacologic treatment	OR	95% CI		P > z
		Lower	Upper	
Male	0.82	0.62	1.09	0.174
Age < 45	0.70	0.49	1.01	0.056
Cardiologist	0.98	0.75	1.28	0.884
South region	0.81	0.61	1.06	0.122
Rural	1.15	0.81	1.65	0.432
Academic practice	1.81	0.89	3.69	0.101
Study arm				
Intervention 1	0.48	0.31	0.74	0.001
Intervention 2	0.87	0.58	1.29	0.476
Round	1.14	0.78	1.66	0.500
Study arm * round				
Intervention 1 * round 2	4.12	2.32	7.31	0.000
Intervention 2 * round 2	2.62	1.52	4.52	0.001
Constant	0.60	0.42	0.85	0.004

(c) Monitoring and follow-up				
Monitoring and follow-up	OR	95% CI		P > z
		Lower	Upper	
Male	0.94	0.70	1.26	0.674
Age < 45	0.72	0.50	1.05	0.085
Cardiologist	0.64	0.48	0.85	0.002
South region	0.78	0.59	1.03	0.084
Rural	1.68	1.16	2.42	0.006
Academic practice	1.47	0.69	3.14	0.322
Study arm				
Intervention 1	1.61	1.04	2.49	0.032
Intervention 2	1.71	1.11	2.64	0.015
Round	1.05	0.68	1.64	0.821
Study arm * round				
Intervention 1 * round 2	2.38	1.31	4.31	0.004
Intervention 2 * round 2	3.52	1.94	6.39	0.000
Constant	0.31	0.21	0.46	0.000

**Table 4:** Use of SGLT2-inhibitors or GLP1 receptors agonists

	Round 1	Round 2	p-value
SGLT2i/GLP1 RA			
Control	35.4%	45.0%	0.082
Int 1	38.4%	66.9%	< 0.001
Int 2	43.3%	72.3%	< 0.001
p-value	0.359	< 0.001	

*SGLT2i* sodium–glucose cotransporter-2 inhibitors, *GLP1 RA* glucagon-like peptide-1 receptor agonists

Doing the same analysis for nonpharmacologic treatment, 27.1% of physicians performed at least half of the items at baseline, with Intervention 1 performing significantly worse compared to the other 2 arms (18.5% versus 31.2%, p < 0.001). When we did the pre-post analysis, the difference-in-difference model showed improvements of + 30.0% by Intervention 1 (p < 0.001) and + 22.8% by Intervention 2 (p = 0.001). These results were robust in the full logistic model (Table 3b).

Conditioning non-pharmacologic treatment on getting the CV risk correct in the pre-post, difference-in-difference model, we found Intervention 1 improved by + 22.5% versus Control (p = 0.016). By contrast, although Intervention 2 trended in the right direction, this group only improved by + 12.4% versus Control, which proved not to be significant (p = 0.213).

Finally, in monitoring and follow-up, which looked at monitoring HgbA1C, blood pressure, and follow-up visit frequency, among other items, the difference-in-difference model showed that Intervention 1 improved by + 20.3% (p = 0.004) and Intervention 2 improved by + 29.8% (p < 0.001), again versus Control. These results again remain significant in the full logistic model (Table 3c).

## Discussion

The generational decline in CVD morbidity and mortality is primarily driven by advances in secondary prevention and risk identification [25]. A core strategy is to use population-based risk scores, such as the 10-year atherosclerotic CVD (ASCVD) risk [26]. These risk scores serve to guide clinical management decisions, especially starting with pharmacologic intervention [27]. However, population risk strategies are of limited utility if they are not used and if they are not done in concert with individual patient risk [28].

Advances in diagnostic testing have made it possible to use advanced protein analysis obtained from a simple blood test done in the office setting to estimate individual CV risk. We conducted the QuiCER DM Study, a randomized controlled trial to determine if a novel diagnostic tool, the *SomaSignal™* CVD-T2D Test, leads to improvement in the ability to risk stratify and treat patients with T2DM.

Our study had four main findings. First, use of the test significantly improved CV risk stratification in both intervention groups. This is important because the test predicts the risk of a CV event within the next 4 years, as opposed to conventional 10-year risk prediction tools. This could lead to more urgent and timely prescribing of evidence-based therapies in patients with T2DM, potentially improving patient outcomes. Second, while we found no differences between the control and intervention groups with respect to overall pharmacologic management, it was heartening to see prescribing SGLT2 inhibitors and GLP1 receptor agonists increased significantly between rounds 1 and 2, especially in cases with high-risk scores. Current evidence-based guidelines support use of these classes of drugs in patients with T2DM due to significant improvements in CV morbidity and mortality [29]. In contrast, while statin ordering overall increased, correctly identifying moderate versus high dose statins in their patients did not increase because of correct CV risk stratification. Current guidelines recommend moderate- or high-intensity statin therapy in diabetic patients with multiple CV risk factors [29, 30]. While we do not have documented reasons for this finding, we know barriers to physician adherence to clinical practice guidelines vary from lack of familiarity with current guidelines to inability to overcome the inertia of previous practice [31–33]. Third, non-pharmacologic treatment, including counseling on nutrition, weight loss, exercise, and smoking cessation, improved significantly in both intervention arms between rounds 1 and 2 compared to control. This aligns with current recommendations to encourage and support diabetes self-management goals [30]. Lastly, monitoring HgbA1C, blood pressure, and follow-up visit frequency improved significantly in both intervention groups as compared with control. While HgbA1c and blood pressure measurements are surrogate markers, we know good control is associated with improved patient outcomes, both microvascular and macrovascular [29].

Interestingly, the Metabolic Panel results given to Intervention 2 did not appear to confer additional benefit over just the CVD-T2D results given to Intervention 1. A possible explanation may be that while the estimated risk score is easily interpreted, i.e., a 4-year risk score with a higher number indicating higher risk of an event, the metabolic panel consists of several items, e.g., likelihood of liver fat, to be interpreted individually. Additionally, there is no evidence to date documenting improved patient outcomes by treating these individual items. Further research is needed to clarify the role of the metabolic panel in the management of patients with T2DM and CV risk factors and whether a metabolic panel alone, absent the CVD-T2D results, would give rise to similar results.

Assessment of CV risk factors and calculation of 10-year risk of ASCVD in patients without known ASCVD (primary prevention) is recommended between the ages of 40 and 75 years of age [34]. A recent Cochrane systematic review showed that providing quantitative CV risk score data to clinicians and patients only had modest effects on levels of CV risk factors and subsequent estimated 10-year CVD risk at follow-up [35]. Providing this information was associated with increased initiation/intensification of lipid-lowering and antihypertensive medications and there was no evidence of harm from quantitative risk assessment.

Optimizing risk factors includes pharmacologic and non-pharmacologic interventions. Our study demonstrated that providing clinicians, both cardiologists and PCPs, with a simple blood test to provide a 4-year risk stratification score increased use of evidence-based pharmacologic treatments in patients with T2DM, namely SGLT2 inhibitors and GLP1 receptor antagonists. Clinicians were also more likely to counsel patients on lifestyle interventions and improved monitoring of HgbA1c and blood pressure.

### Limitations

Our study has some limitations. This study focused on intermediate and high CV risk. We do not know if patients at low CV risk would benefit from this test. Data were derived from the care of simulated patients. However, multiple studies have shown that the results from simulated patient studies using the validated tool are the same as results from similarly conducted studies in real-world patients. This suggests use of simulated patient studies may accelerate the evaluation of clinical utility [36, 37]. Whether these improvements in care due to the CVD-T2D test will translate to real world patients remains to be demonstrated. Lastly, we focused on clinical practice change not long-term patient outcomes.

## Conclusion

The results of this randomized controlled trial show that use of the CVD-T2D test significantly improved CV risk stratification for T2DM patients, a crucial step in accurate prediction and prevention of CVD morbidity and mortality. While the test did not demonstrate improvements in overall pharmacologic management in the CPV scores, the test influenced important care decisions, as evidence by statistically significant increases in use of evidence-based pharmacologic treatments, specifically SGLT2 inhibitors and GLP1 receptor antagonists and recommendation of evidence-based non-pharmacologic treatments for patients with T2DM. Future research should seek to further understand benefits of the CVD-T2D tool and the potential for out-of-date CVD care strategies to effect the impact of accurate risk stratification on overall CVD pharmacologic management.

## Supplementary Information

**Additional file 1: Table S1.** Matrix of the nine simulated patient cases used in the study.

**Additional file 2.** Educational material on CVD burden and introduction of the novel diagnostic test that predicts the absolute risk of cardiovascular events in patients with type 2 diabetes mellitus.

**Additional file 3.** Educational factsheet on cardiovascular disease risk in type 2 diabetes and summary of the novel diagnostic test with metabolic factors.

## Data Availability

Data available upon reasonable request to the corresponding author.
