# Supplementary material for: Clinical utility of a novel test for assessing cardiovascular disease risk in type 2 diabetes: a randomized controlled trial
Source: Diabetol Metab Syndr. 2023 Jul 13;15:155. doi: 10.1186/s13098-023-01122-w (PMC10339653; doi:10.1186/s13098-023-01122-w)
Supplement: Supplementary file 1 — Additional file 1: Table S1. Matrix of the nine simulated patient cases used in the study. [file 13098_2023_1122_MOESM1_ESM.docx]

Table S1. Matrix of the Nine Simulated Patient Cases used in the Study

|  | **Variant A** | **Variant B** | **Variant C** |
| --- | --- | --- | --- |
|  | *High clinical risk* | *Intermediate clinical risk* | *High clinical risk* |
| **T2DM with**  **3 or more CV Risk Factors** | 67/F Caucasian  Comes in the office for pain on the left shoulder after being lost to follow-up  Risk factors: Hypertension, dyslipidemia, smoking  Estimated clinical ASCVD risk: 23·9%  Estimated CVD-T2D Score: 96 | 52/M Caucasian  Comes in for his annual wellness visit  Risk factors: Hypertension, dyslipidemia, obesity  Estimated clinical ASCVD risk: 8·1%  Estimated CVD-T2D Score: 75 | 69/F Asian American  Comes in the office for routine DM follow up care  Risk factors: Hypertension, dyslipidemia, obesity  Estimated clinical ASCVD risk: 22·9%  Estimated CVD-T2D Score: 14 |
| **T2DM with CKD** | 58/F Black  Routine follow up care  Risk factors: CKD St 3, Hypertension, Dyslipidemia  Estimated clinical ASCVD risk: 27·7%  Estimated CVD-T2D Score: 95 | 47/M Black  Routine DM care; on RoS has muscle aches on high-intensity statin  Risk factors: CKD St 4, Hypertension, Dyslipidemia  Estimated clinical ASCVD risk: 13·6%  Estimated CVD-T2D Score: 75 | 67/F Caucasian  Follow up DM care  Risk factors: CKD St 3, Hypertension, Dyslipidemia, former smoker  Estimated clinical ASCVD risk: 20·6% Estimated CVD-T2D Score: 6 |
| **T2DM with**  **2 CV risk**  **factors** | 65/M Black  Comes in for his annual wellness visit  Risk factors: Hypertension, dyslipidemia  Estimated clinical ASCVD risk: 24·5%  Estimated CVD-T2D Score: 97 | 66/F Asian American  Consult due to dizziness with SGLT2i; A1C 10·2%  Risk factors: Hypertension, dyslipidemia  Estimated clinical ASCVD risk: 18·9%  Estimated CVD-T2D Score: 58 | 48/M Caucasian  Routine follow up visit for BPH; reports nausea with GLP-1 RA  Risk factors: Dyslipidemia, smoking  Estimated clinical ASCVD risk: 20·1% Estimated CVD-T2D Score: 18 |
