# Supplementary material for: Clinical utility of a novel test for assessing cardiovascular disease risk in type 2 diabetes: a randomized controlled trial
Source: Diabetol Metab Syndr. 2023 Jul 13;15:155. doi: 10.1186/s13098-023-01122-w (PMC10339653; doi:10.1186/s13098-023-01122-w)
Supplement: Supplementary file 2 — Additional file 2. Educational material on CVD burden and introduction of the novel diagnostic test that predicts the absolute risk of cardiovascular events in patients with type 2 diabetes mellitus. [file 13098_2023_1122_MOESM2_ESM.pdf]

Lowering the CVD Burden by Using a Simple Diagnostic Test that Predicts the Absolute Risk of Cardiovascular Events in Patients with Type 2 Diabetes Mellitus

The SomaSignal CVD-T2D Risk Test

The Health and Economic Burden of Cardiovascular Disease (CVD)

Health Outcomes	Costs	Clinical Presentation
• Leading cause of morbidity and mortality worldwide<br>• Affects approximately 110 million people in the US	• Total medical costs are over $600 billion<br>• Increases in cost over time expected to be highest in Hispanics and people aged 80 and above	• Massive variation in presentation<br>• Often asymptomatic<br>• Clinical presentation of CV risk may not align precisely with actual risk

QURE HEALTHCARE

© QURE 2022

The Challenge of Cardiovascular Disease in Patients with Type 2 Diabetes (T2DM)

1

- T2DM increases the risk of **fatal** and **non-fatal** cardiovascular (CV) events
- Control of multiple CV risk factors in T2DM patients can **decrease** CV events and death by **half**

2

- T2DM and CV events have **overlapping risk factors**, making CVD risk assessment difficult
- Pinpointing the likelihood of when CVD will occur in T2DM patients is especially challenging

3

- This control is not easily achieved – more than half of T2DM patients do **not** reach their target

QURE HEALTHCARE

Martín-Timón, I., Sevillano-Collantes, C., Segura-Galindo, A., & Del Cañizo-Gómez, F. J. (2014). Type 2 diabetes and cardiovascular disease: Have all risk factors the same strength?. *World journal of diabetes*, *5*(4), 444–470. https://doi.org/10.4239/wjd.v5.i4.444

© QURE 2022

Multiple Overlapping Risk Factors in T2DM Patients Compound Asymptomatic CVD Risk

Failure to control one risk factor is associated with poor control of other risk factors.

CVD is higher in patients with mild kidney disease than in patients without evidence of kidney disease.

Comprehensive control of multiple cardiovascular risk factors in high-risk patients is suboptimal worldwide.

Large benefits are seen when multiple cardiovascular risk factors are addressed simultaneously.

QURE HEALTHCARE

Levin A. Clinical epidemiology of cardiovascular disease in chronic kidney disease prior to dialysis. Semin Dial. 2003 Mar-Apr;16(2):101-5. doi: 10.1046/j.1525-139x.2003.16025.x. PMID: 12641872.
American Diabetes Association. 10. Cardiovascular disease and risk management: Standards of Medical Care in Diabetesd2021. Diabetes Care 2021;44(Suppl.1):S125–S150

© QURE 2022

Need for a Diagnostic Tool that Stratifies CV Risk for Secondary Prevention in T2DM Patients

The ACC/AHA Recommended ASCVD Risk Score

- Targets patient risk as a **primary prevention** method
- Determines risk on the basis of selective clinical features
- Produces results as long-term patient health outcomes
- Not optimized for patients with diabetes or other comorbidities

The CVD-T2D Risk Test

- Targets patient risk as a **secondary prevention** method
- Validated in a broad population across socioeconomic and ethnicities with multiple comorbidities to give an **individualized and patient-specific score**
- Median time to event is **1.7 years** across all patients, allowing time for effective risk reduction
- Key for **asymptomatic** patients

QURE HEALTHCARE

Blom DJ, et. al. The challenge of multiple cardiovascular risk factor control outside Western Europe: Findings from the International Cholesterol management Practice Study. European Journal of Preventive Cardiology. 2020, Vol. 27(13) 1403–1411
American Diabetes Association. 10. Cardiovascular disease and risk management: Standards of Medical Care in Diabetesd2021. Diabetes Care 2021;44(Suppl.1):S125–S150
Byyny R. L., social determinants of health: reforming education and public health to improve health in the United States. The Pharos.. Alpha Omega Alpha Honor Medical Society. 2017.

© QURE 2022

SomaLogic’s New Diagnostic Techniques and Technology

Slow Off-rate Modified Aptamers (SOMAmer reagents): specific protein binding reagents

- Combines thousands of different SOMAmer reagents to measure thousands of proteins in a **single blood sample**
- Uses cutting-edge analytics with artificial intelligence & machine learning to produce precise, personalized tests

Results in the
Cardiovascular Disease in Type 2 Diabetes (CVD-T2D) Risk Score

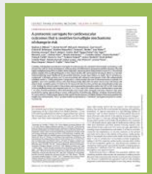

“A proteomic surrogate for cardiovascular outcomes that is sensitive to multiple mechanisms of change in risk”
- A test that predicts major cardiovascular outcomes and deaths in a time scale consistent with clinical outcome trials
Williams et al., Sci. Transl. Med. 14, eabj9625 (2022)

© QURE 2022

The ACC/AHA Recommended ASCVD Risk Score

Assesses CVD Risk by Accounting for:

Age	Sex
Race	Cholesterol Level
Blood Pressure Level	Medical History

- Estimates the patient's **10-year** ASCVD risk at an initial visit to establish a reference point.
- Forecasts the potential impact of different interventions on patient risk.
- Reassess ASCVD risk at follow-up visits.
- Should be used for **primary prevention patients (those without ASCVD) only.**
- Not optimized for patients with diabetes or other comorbidities.

QURE HEALTHCARE

© QURE 2022

The Clinically Validated CVD-T2D Risk Score

Estimates the **absolute risk** of having a CV event within **4 years** for patients over 40 with type 2 diabetes, with or without CVD or CKD

- Using only a single blood sample
- 2 weeks from the time sample arrives

Predicts the incidence of **myocardial infarction, stroke, hospitalization for heart failure, or all-cause death**

Median time of 1.7 years to a CV event for all risk categories

- Exposes risk not identified using traditional methods
- Risk stratify and act in a **shorter amount of time**
- Sufficiently long to implement therapeutic changes
- Not so distant that risk becomes deniable

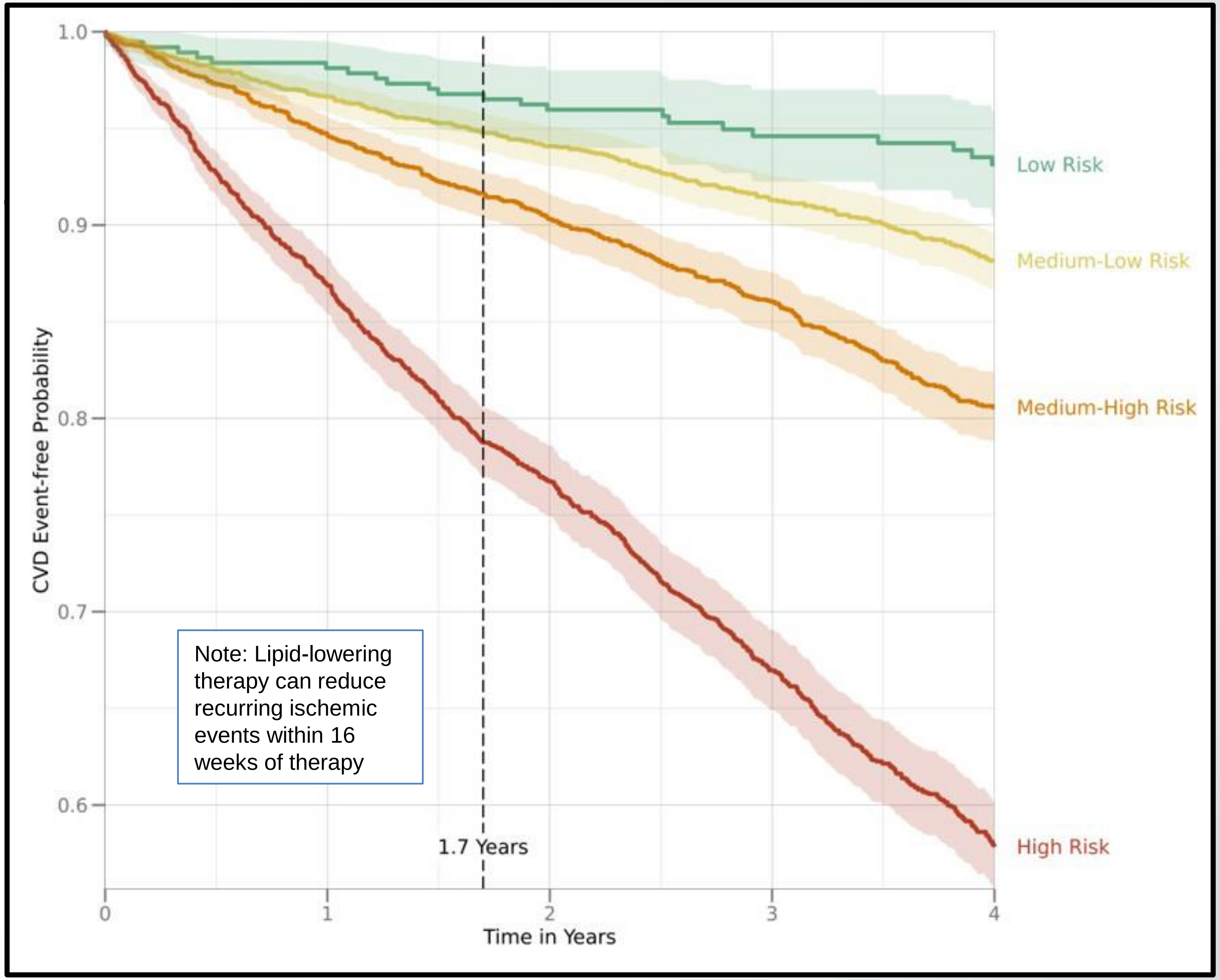

Ganz, P. et al. Development and Validation of a Protein-Based Risk Score for Cardiovascular Outcomes Among Patients With Stable Coronary Heart Disease. JAMA 315, 2532-2541 (2018).

Eagle KA, Ginsburg GS, Musunuru K, et al. Identifying patients at high risk of a cardiovascular event in the near future: current status and future directions: report of a national heart, lung, and blood institute working group. Circulation. 2010;121(12):1447-1454.

Schwartz GG, Olsson AG, Ezekowitz MD, et al. Effects of Atorvastatin on Early Recurrent Ischemic Events in Acute Coronary Syndromes: The MIRACL Study: A Randomized Controlled Trial. *JAMA.* 2001;285(13):1711–1718. doi:10.1001/jama.285.13.1711

QURE HEALTHCARE

© QURE 2022

The SomaSignal CVD-T2D Risk Score Delivers Easy to Interpret Results

CARDIOVASCULAR DISEASE IN TYPE 2 DIABETES

TEST	RISK SCORE	REPORTABLE RANGE	RISK CATEGORY
Risk of Cardiovascular Event in 4 Years<br>R1140	60%	0-100%	0% 7.5% 25% 50% 100%<br>LOW, MEDIUM-LOW, MEDIUM-HIGH, **HIGH**

Quantifies risk on a measurable scale with a singular risk score

Provides information for action, e.g., initiation of GLP1-inhibitor, SGLT2 inhibitor, and statins to reduce risk of secondary events

For patients without known cardiovascular risk	For patients with higher cardiovascular risk or established ASCVD[4]
Use of metformin and implementation of comprehensive lifestyle changes is recommended, including weight management and physical activity.	Consider either GLP-1 Receptor Agonist or SGLT2 inhibitor with proven CVD benefit.

Metabolic Factors Bundled Results

Additionally, the CVD-T2D risk test is coupled with the Metabolic Factors Panel which provides a quick snapshot of key metabolic results to improve diagnosis and treatment pathways

CARDIOMETABOLIC FACTORS

TEST	RESULT	REPORTABLE RANGE	CHART
Liver Fat	Some Excess Fat	N/A	NO EXCESS FAT / **SOME EXCESS FAT**
Glucose Tolerance	Normal Tolerance	N/A	**NORMAL TOLERANCE** / IMPAIRED TOLERANCE
Kidney Function	94 ml/min/1.73m^2	5-100 ml/min/1.73m^2	80, 60, 40, 20; **HIGH** / LOW
Alcohol Impact	Lower Impact	N/A	**LOWER IMPACT** / HIGHER IMPACT
Cardiorespiratory Fitness – VO$_2$ Max	26.4 ml/kg/min	15.0-85.0 ml/kg/min	EXCELLENT / GOOD / **FAIR** / LOW
Resting Energy Rate	1843 calories per day	500-3500 cal/day	1000, 1500, 2000, 2500, 3000
Body Fat Percentage	53%	3-80%	LESS / AVERAGE / **MORE**
Lean Body Mass	98.6 lbs	20.0-100.0 lbs	**MORE** / **AVERAGE** / LESS
Visceral Fat	3.88 lbs	0.5-12.5 lbs	LESS / AVERAGE / **MORE**

QURE HEALTHCARE

© QURE 2022

Benefits of CVD-T2D Bundled Risk Assessment

- Rapidly assesses a blood draw to provide an objective assessment of CVD risk within the next four years for T2DM patients
- Exposes risk not identified using traditional methods
- Treat co-morbidities to mitigate risk
- Consolidated list of key metabolic measures

© QURE 2022
