# Supplementary material for: Clinical utility of a novel test for assessing cardiovascular disease risk in type 2 diabetes: a randomized controlled trial
Source: Diabetol Metab Syndr. 2023 Jul 13;15:155. doi: 10.1186/s13098-023-01122-w (PMC10339653; doi:10.1186/s13098-023-01122-w)
Supplement: Supplementary file 3 — Additional file 3. Educational factsheet on cardiovascular disease risk in type 2 diabetes and summary of the novel diagnostic test with metabolic factors. [file 13098_2023_1122_MOESM3_ESM.pdf]

Cardiovascular Disease in Type 2 Diabetes (CVD-T2D) with Metabolic Factors

somalogic™

Estimating Risk in Cardiovascular Disease (the ASCVD risk score)

- A primary prevention tool that determines risk on the basis of selective clinical features
- Not optimized for patients with diabetes or other comorbidities

The CVD-T2D Test is a reliable, sensitive, and affordable diagnostic technology based on the identification of a protein-based analysis.

A diagnostic blood test that assesses cardiovascular & metabolic risks:

- ✓ Cardiovascular risk in patients with type 2 diabetes over a 4-year time range
- ✓ Likelihood of excess liver fat
- ✓ Likelihood of having impaired glucose tolerace
- ✓ Estimated Glomerular Filtration Rate (eGFR)
- ✓ Weekly alcohol impact
- ✓ Peak exercise capacity
- ✓ Resting energy expenditure
- ✓ Percentage of total body fat
- ✓ Amount of visceral fat, lean body mass

- A **single** blood sample
- **Secondary prevention** tool
- **Exposes risk** not identified using traditional methods
- Key for **asymptomatic patients**
- Produces **precise and personalized** test results

Indication for Use

- A blood-based test you can use to determine cardiovascular risk (MI, stroke, HF-related hospitalization, and all-cause mortality) in patients with T2DM

Stratifies patient's CV risk for secondary prevention to guide physicians in streamlining interventions to achieve targets

- The CVD-T2D Risk Test has been validated in a broad population across socioeconomic and ethnicities with multiple comorbidities to give you an **individualized and patient-specific score**
- The CVD-T2D Risk Test gives you an event time (median) of **1.7 years**, giving you and your patient time for effective risk reduction - Williams et al., Sci. Transl. Med. 14, eabj9625 (2022)

Patient & Provider Benefits

- Assesses overlapping risk measures of CV and metabolic disorders
- Objective assessment of CVD risk within the next four years
- Identifies treatment regimens that best match patient needs
- Provides a listing of key metabolic measures that could play a role in CV risk

Sample Collection and Turn Around Time

- Blood sample, shipped frozen from your office
- Turnaround time: 2 weeks from the time sample received

Easy-to-Read Test Results with Evidence Based Recommendations

Test	Risk Score	Risk Category
Risk of Cardiovascular Event in 4 Years	60%<br>(0-100%)	LOW / MED-LO / MED-HI / HIGH<br>0% 7.5% 25% 50% 100%
Kidney Function	94<br>ml/min/1.73 m²<br>(5-100 ml/min/1.73 m²)	HIGH<br>80% 60% 40% 20%

Delivers a singular and measurable risk score
Quantifying risk on an actionable scale

For patients w/o known CVD Risk

Use of metformin and implementation of comprehensive lifestyle changes is recommended, including weight management and physical activity

For patients w/ higher CV risk as established by the CVD-T2D Score

Add either GLP-1 Receptor Agonist or SGLT2 inhibitor for proven CVD benefit
